# Study on the differences of phyllosphere microorganisms between poplar hybrid offspring and their parents

**DOI:** 10.7717/peerj.12915

**Published:** 2022-03-15

**Authors:** Changjun Ding, Weixi Zhang, Yanbo Wang, Mi Ding, Xiaojiang Wang, Aiping Li, Dejun Liang, Xiaohua Su

**Affiliations:** 1 State Key Laboratory of Tree Genetics and Breeding, Research Institute of Forestry, Chinese Academy of Forestry, Beijing, China; 2 Key Laboratory of Tree Breeding and Cultivation of State Forestry Administration, Research Institute of Forestry, Chinese Academy of Forestry, Beijing, China; 3 Inner Mongolia Academy of Forestry Sciences, Hohhot, Inner Mongolia, China; 4 Liaoning Provincial Poplar Institute, Gaizhou, Liaoning, China

**Keywords:** *Populus simonii* ‘DH4’, *P. nigra* ‘DH5’, Phyllosphere microganism, Parent, Hybrid

## Abstract

The females and males of dioecious plants have evolved sex-specific characteristics in terms of their morphological and physiological properties. However, the differentiation of phyllosphere microorganism of dioecious plants between parents and hybrid offspring remain largely unexplored. Here, the phyllosphere bacterial and fungal community diversity and composition of female (*Populus nigra* ‘DH5’ (PNDH5)), male (*P. simonii* ‘DH4’ (PSDH4)), and the hybrid offspring (*P. simonii* × *P. nigra* ‘DH1’ (PSPNDH1), *P. simonii* × *P. nigra* ‘DH2’ (PSPNDH2), *P. simonii* × *P. nigra* ‘DH3’ (PSPNDH3)) were investigated using 16S rDNA/ITS rDNA gene-based Illumina NovaSeq 6000 sequencing. There was considerable variation of plant height, diameter at breast height, leaf area, length of petioles, leaf moisture content, and starch among different samples, and PSDH2 owned the highest plant height, diameter at breast height, and length of petioles. No distinct differences of phyllosphere bacterial community diversity were observed among PSDH4, PNDH5, PSPNDH1, PSPNDH2, and PSPNDH3; while, PSPNDH2 owned the highest fungal Pielou_e index, Shannon index, and Simpson index. Firmicutes and Ascomycota were the predominant phyllosphere bacterial and fungal community at the phylum level, respectively. Bacilli and Gammaproteobacteria were the two most dominant bacterial classes regardless of parent and the hybrid offspring. The predominant phyllosphere fungal community was Dothideomycetes at the class level. The NMDS demonstrated that phyllosphere microbial community obviously differed between parents and offspring, while the phyllosphere microbial community presented some similarities under different hybrid progeny. Also, leaf characteristics contributed to the differentiation of phyllosphere bacterial and fungal communities between parents and hybrid offspring. These results highlighted the discrimination of phyllosphere microorganisms on parent and hybrid offspring, which provided clues to potential host-related species in the phyllosphere environment.

## Introduction

Poplar is an important forest tree species in temperate regions of the world, with strong adaptability and wide geographical distribution (Hou, 2021), which is often chosen as artificial forestation in our country (Zhang, Li & He, 2006). Due to its rapid growth rate, good wood quality, and excellent disease resistance, poplar plays an important role in wood production, forest construction, landscaping, and ecological protection (Aylott et al., 2010). So far, the genetically improved poplar varieties used and popularized in forestry production in China are mainly cultivated through artificial hybridization (Zhang, Li & He, 2006). In comparison with their parents, interspecific hybrids have stronger fecundity, growth vigor, superior adaptability, stress resistance and other functional traits, which achieved outstanding economic benefits (Xu et al., 2019). For example, *Populus euramericana* (*P. deltoides × P. nigra*) produces large quantities of industrial wood and performs strong heterosis in Asia, Europe and America (Zhang & Li, 2003; Stettler et al., 1996). *P. simonii × P. nigra* is widely planted for timber forest construction and shelter in cold and dry regions due to its high cold tolerance (FAO, 2014). At present, the formation mechanism of poplar heterosis has been extensively explored from the perspective of growth traits (Ren et al., 2020; Li et al., 2013; Zhang et al., 2011), photosynthetic capacity (Dong et al., 2016), water use efficiency, hormone contents, physiology variation (Kanaga et al., 2008; Wang et al., 2011b), genetic relationship between hybrid offspring and parents (Jiang et al., 2004; Zanewich, Pearce & Rood, 2018; Su, Ding & Ma, 2010), as well as abiotic stresses (Wang et al., 2009). Recently, the differences between parents and hybrids have become a rather interesting topic. *P. simonii* is characterized by small diamond-shaped oval leaves, which are resistant to cold, drought and barrenness and are widely used in the construction of shelterbelt and timber forest in barren land, arid and cold regions of northern China (Cheng et al., 2019). *P. nigra* is an important economic species in the alluvial plain area of Europe, which is characterized by large and triangular leaves, rapid growth growing, and high-yield production (Storme et al., 2004). Therefore, the hybridized combinations of excellent clones of *P. simonii* and *P. nigra* show excellent wood performance, fast growth, strong cold resistance, and drought tolerance (Zhou et al., 2019), which are generally distributed in the northern region of China. The early research on *P. simonii × P. nigra* mainly focused on the introduction and cultivation of germplasm resources (Ren et al., 2006). In recent years, the studies have mainly focused on the use of transgenic technology to enhance growth and photosynthetic characteristics (Zhao et al., 2015), salt tolerance (Bai et al., 2006), disease resistance (Wang, 2007), insect resistance (Lin et al., 2006), and genomic information, as well as molecular characterization of stress tolerance and breeding (Wang et al., 2011a). However, the differences of phyllosphere microbes between parents and hybrids have received relatively less attention. Moreover, whether the differences of plant functional traits could drive the divergence of phyllosphere bacterial and fungal communities, particularly in parents and their hybrid offspring, is still unclear.

The phyllosphere, as the interface between the aboveground parts of plants and air (Berg et al., 2016), harbors more than 10^26^ microorganisms (Vorholt, 2012), such as bacteria, fungi, and archaea (Delmotte et al., 2009), which are called phyllosphere microbes (Lindow & Leveau, 2002). Phyllosphere microbiota has been found to be in symbiosis with hosts and affects the growth and ecological function of the host in many ways, such as influencing the fitness and development through production of growth-promoting nutrients (Bulgarelli et al., 2013) and phytohormones (Sha et al., 2017), increasing plants stress tolerance (Kaczmarczyk et al., 2011), and protection of hosts against pathogen (Carrion et al., 2019; Innerebner, Knief & Vorholt, 2011). Therefore, it is of great ecological significance to understand the diversity and composition of phyllosphere microbial communities. However, systematic comparisons on phyllosphere microorganisms of parent and the hybrid offspring are still lacking.

Plant species are the main factors affecting the composition of phyllosphere microbial communities (Redford & Fierer, 2009; Whipps et al., 2008; Bodenhausen, Horton & Bergelson, 2013), and different plants were colonized by different microorganisms. The leaf characteristics, including leaf moisture, leaf thickness, mesophyll thickness, phosphorus content, carbohydrates, amino acids, phenolic, and organic acids secreted mainly by leaves, are limiting factors during microbe colonization in the phyllosphere (Rastogi et al., 2012; Muller & Ruppel, 2014). Hunter et al. (2010) revealed that plant morphology and soluble carbohydrate content significantly influenced the diversity of the phyllosphere bacterial community, while the effects of plant functional traits on phyllosphere microbial community need to be further addressed. Hence, further investigation of the response of phyllosphere microbial communities to parent and the hybrid offspring could provide valuable leads for how crossbreeding shapes the phyllosphere microbial communities.

*P. simonii* × *P. nigra* is a major cultivated variety which has been generalized in a broad region of northeast China due to its outstanding stress resistance and environmental adaptability under desertification conditions and with the improvement of the ecological environment. In this study, with the aim of obtaining a high growth rate and highly resistant offspring, *P. simonii* ‘DH4’ (female) and *P. nigra* ‘DH5’ (male) were selected as parents, and *P. simonii* × *P. nigra* ‘DH2’, *P. simonii* × *P. nigra* ‘DH3’, *P. simonii* × *P. nigra* ‘DH1’ were chosen as three hybrid offsprings. This work addressed two key questions: (і) whether there were differences in the phyllosphere bacterial and fungal communities under different hybrid offspring as well as their parent, (ii) do leaf characteristics contribute to the differentiation of phyllosphere bacterial and fungal communities between parents and hybrid offspring.

## Materials and Methods

### Experimental site and experimental materials

The experimental field site was located in Baituliang Forest Farm, Dalat Banner, Erdos City, Inner Mongolia Autonomous Region (37°35′24″∼407°35′247°N, 106°42′40″∼111°35′247°E), which is classified as temperate continental monsoon climate. The annual average temperature is 6.8 °C with a minimum of −22 °C in winter and a maximum of 37 °C in summer. The annual precipitation and annual average frost-free period are 300 mm and 150 days, respectively.

In the spring of 2015, F1 progeny seeds of *P. simonii* × *P. nigra* ‘DH1’ (PSPNDH1), *P. simonii* × *P. nigra* ‘DH2’ (PSPNDH2), and *P. simonii* × *P. nigra* ‘DH3’ (PSPNDH3) were obtained from the hybridizations between female clone of *P. simonii* ‘DH4’ and male clone of *P. nigra* ‘DH5’ and planted in the greenhouse of CAF (Chinese Academy of Forestry), Beijing, China. In the spring of 2016, cutting seedlings of F1 progeny and their parents were planted with the density of 30 cm × 50 cm at Baituliang Forest Farm, Dalat Baner, Inner Mongolia Autonomous Region. In the spring of 2018, 16 plants for each genotype of F1 progenies and each clone of parents were grown in a plot with a spacing of 10 × 15 m. A total of 25 plots were designed and five replicates were randomly distributed (Fig. S1). Plants were planted in each plot with the density of 2 m × 3 m.

### The determination of plant functional traits

The height was measured with an altimeter. The diameter at breast height was measured using a breast diameter ruler to measure the diameter of the tree trunk 1.3 m above the ground. The relative moisture content of leaf was weighed by the drying method. The length of petiole was measured with a ruler. Leaf area was calculated using Image J and leaves were scanned with a CanonScan LiDE 210 (Canon Inc., Tokyo, Japan). The contents of soluble sugar and starch were determined by anthrone colorimetry.

### Sample collection

A total of 15–20 leaves from the tip of the shoots in the middle of the canopy from three directions, including 120° around the tree, were collected, and leaves from ten trees at each plot were mixed as one replicate. A total of 25 leaf samples (5 clones × 5 replicates) were collected. To analyze the phyllosphere microbial community on the leaves, 10 g of leaves from each replicate were cut into pieces and submerged in phosphate-buffered solution (20 mL, PBS, 0.01 M, pH 7.4) (leaf weight/volume TE buffer = 1:20). After vigorous shaking on a shaker at 200 r/min for 30 min at room temperature, leaves were removed, and the suspension containing phyllosphere microorganism was retained. The suspension was filtered through sterile vacuum filtration, and phyllosphere microbes from the oscillating liquid were collected on a 0.22 µm filter membrane and then placed into 2 ml sterile centrifuge tubes. The samples were stored at −80 °C prior to DNA extraction and high-throughput sequencing.

### High-throughput sequencing

According to the manufacturer’s instructions, we used the FastDNA SPIN Kit for soil (MP Biomedical, Santa Ana, CA, USA) to extract Genomic DNA from the filter. The NanoDrop ND-1000 spectrophotometer (Thermo Fisher Scientific, Waltham, MA, USA) was used to measure the DNA concentration (Deng et al., 2021). The primer pairs 338F (5′-ACTCCTACGGGAGGCAGCAG-3′) and 806R (5′-GGACTACHVGGGTWTCTAAT-3′) with barcode sequence were used to amplify the V3–V4 regions of bacterial 16S rDNA gene (Deng et al., 2021). The primer pairs ITS1F (5’-CTTGGTCATAGGAAGAAGTAA-3’) and ITS2 (5’-GCTGCGTTCTTCAGATGC-3’) with barcode sequence were used to amplify the ITS1 region of the fungal ITS rDNA gene. All the PCR reactions were performed with 25 μl mixture, including DNA Template (40–50 ng) 2 μl, 0.25 μl (5 U/μl) of Q5 High-Fidelity DNA Polymerase, 2 μl of dNTPs (2.5 mM), 1 μl (10 uM) of forward and reverse primer, severally; 5 μl of Q5 High-Fidelity GC buffer (5×), 8.75 μl of ddH_2_O, 5 μl of Q5 reaction buffer (5×). The PCR thermal cycling condition was consistent with the study from Deng et al. (2020). Agencourt AMPure Beads (Beckman Coulter, Indianapolis, IN, USA) was used to purify the PCR amplicons, and PicoGreen dsDNA Assay Kit (Invitrogen, Carlsbad, CA, USA) was used to further quantify the PCR amplicons (Deng et al., 2020). PCR products for sequencing were carried out by an Illumina NovaSeq 6000 sequencing platform at Shanghai Personal Biotechnology Co., Ltd., Shanghai, China. The high-throughput sequencing raw data of phyllosphere bacteria and fungi were uploaded in the NCBI database with the SRA accession number PRJNA736812.

### Statistical analysis

The high-quality sequences were finally obtained after removing primers, quality filter, denoise, joint and removal of chimeras (Callahan et al., 2016). And sequences with ≥97% similarity were assigned to the same OUT (Edgar, 2010). Then, the qiime feature-table rarefy function was used for OTU leveling, and the leveling depth was set as 95% of the minimum sample sequence quantity (Kemp & Aller, 2004). Multiple-sample comparisons using one-way analysis with Kruskal–Wallis tests were used to explore the differences of phyllosphere microbial community diversity. Venn diagrams were constructed to show the shared and unique OTUs using RStudio with vegan package (Chen & Boutros, 2011). Linear discriminant analysis effect size (LEfSe) with the Kruskall–Wallis test was employed to identify the bacterial and fungal taxonomic from the phylum to genus levels responsible for the community differentiation between treatments (Segata et al., 2011). The threshold on the logarithmic LDA score for discriminative features was set at 2. For β-diversity, non-metric multidimensional scaling (NMDS) analysis was performed to reveal the differences phyllosphere bacterial (based on weighted-unifrac) and fungal (based on bray distance) community compositions among different samples using RStudio with vegan package (Anderson, 2001). Spearman’s rank correlation was used to analyze the relationships between plant functional traits and phyllosphere microbial community diversity and composition using IBM SPSS (Clarke, 2010).

## Results

### Plant functional traits analysis

There was considerable variations of plant height (*F* = 46.57, *P* < 0.01), diameter at breast height (*F* = 53.98, *P* < 0.01), leaf area (*F* = 35.83, *P* < 0.01), length of petioles (*F* = 287.89, *P* < 0.01), leaf moisture content (*F* = 8.24, *P* < 0.01), and starch (*F* = 3.09, *P* = 0.04) among different samples, and PSDH2 owned the highest plant height, diameter at breast height, and length of petioles with 6.38 m, 5.92 cm, 4.81 cm, respectively. PSDH1 owned the highest starch content with 129.54 mg/g, followed by PSDH2, PSDH3, PSDH4, and PSDH5 (Table 1), while no obvious differences were obtained among different samples (*F* = 1.77, *P* = 0.18) (Table 1).

**Table 1:** Plant functional traits among different samples.

	PSPNDH1	PSPNDH2	PSPNDH3	PSDH4	PNDH5	*F*	*P*
Plant height (m)	5.88 ± 0.25bA	6.38 ± 0.56aA	5.86 ± 0.37bA	3.98 ± 0.19cB	3.88 ± 0.56cB	46.57	<0.01
Diameter at breast height (cm)	4.80 ± 0.68bB	5.92 ± 0.72aA	5.27 ± 0.34bAB	3.45 ± 0.36cC	1.85 ± 0.25dD	53.98	<0.01
Leaf area (mm^2^)	3,806.83 ± 569.79	4,444.29 ± 470.36	4,508.43 ± 469.00	2,964.89 ± 326.61	2,520.19 ± 336.00	35.83	<0.01
Length of petioles (cm)	4.44 ± 0.24bB	4.81 ± 0.20aA	4.76 ± 0.18aA	1.55 ± 0.18dD	3.59 ± 0.35cC	287.89	<0.01
Leaf moisture content (%)	84.59 ± 0.33bABC	87.65 ± 0.13aA	85.59 ± 0.27abAB	83.40 ± 1.60bcBC	81.28 ± 2.75cC	8.24	<0.01
Soluble sugar (mg/g)	134.98 ± 7.34abA	148.86 ± 13.33aA	139.12 ± 12.95abA	145.93 ± 9.81abA	131.72 ± 15.52bA	1.77	0.18
Starch (mg/g)	129.54 ± 45.69aA	104.27 ± 20.47abAB	94.86 ± 17.42bAB	89.32 ± 12.60bAB	78.56 ± 6.58bB	3.09	0.04

**Notes:**

PSPNDH1: *P. simonii* × *P. nigra* cv. ‘DH1’, PSPNDH2, *P. simonii* × *P. nigra* cv. ‘DH2’, PSPNDH3: *P. simonii* × *P. nigra* cv. ‘DH3’, PSDH4: *P. simonii* ‘DH4’, PNDH5: *Populus nigra* ‘DH5’. Average value ± standard deviation (5).

Different lowercase letters and capital letters in each row represent significant difference at *p* < 0.05 and *p* < 0.01, respectively.

### Phyllosphere microbial community diversity

**Figure 1:**
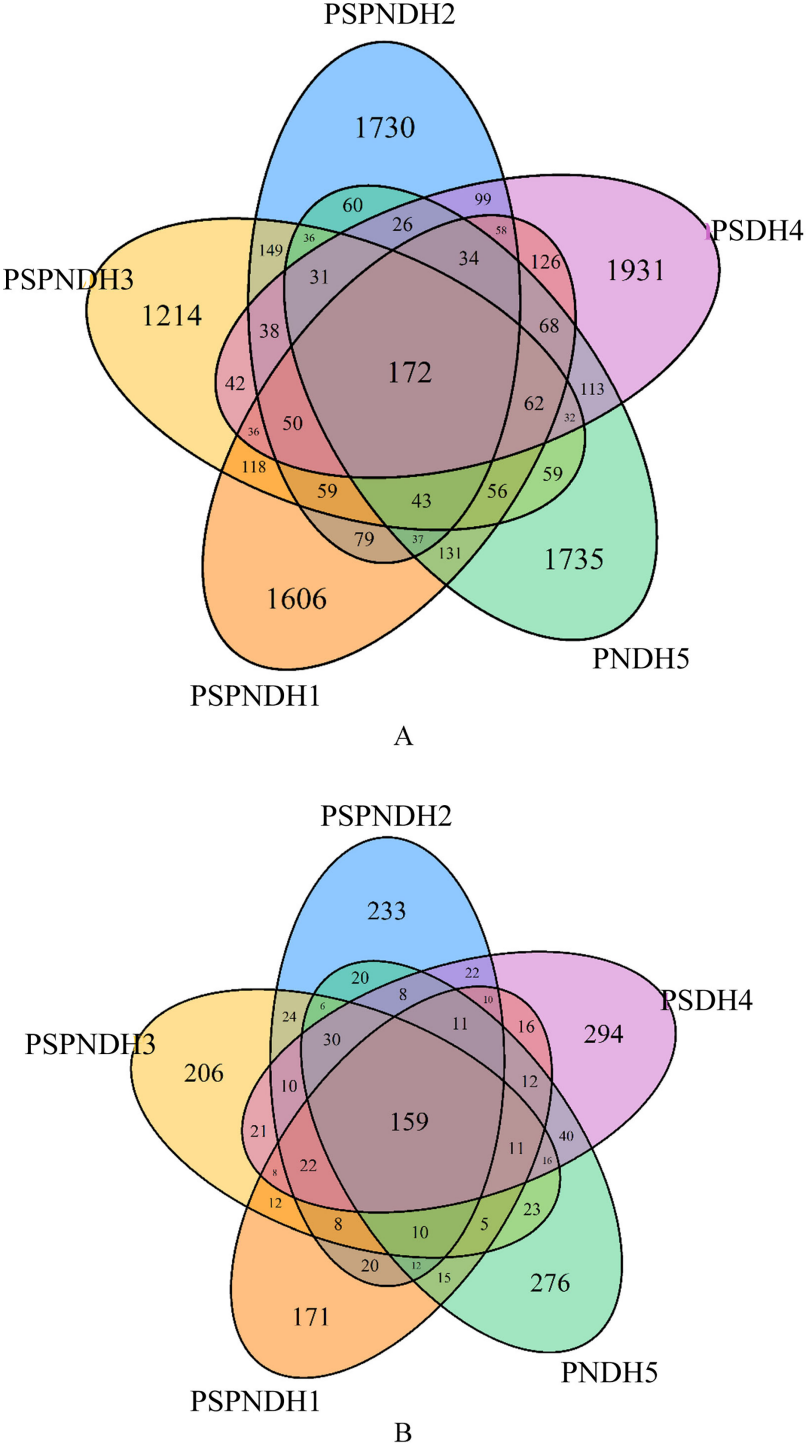
Venn diagram of the phyllosphere bacterial (A) and fungal (B) shared and unique OTUs. PSPNDH1: *P. simonii* × *P. nigra* cv. ‘DH1’, PSPNDH2, *P. simonii* × *P. nigra* cv. ‘DH2’, PSPNDH3: *P. simonii* × *P. nigra* cv. ‘DH3’.

A total of 2,147,073 high quality bacterial sequences were obtained with average sequences of 85,882, which were clustered into 10,031 OTUs. The number of OTUs under PSPNDH2, PSPNDH3, PSPNDH1, PSDH4, and PNDH5 was 2,701, 2,197, 2,735, 2,981, and 2,695, respectively, and the number of unique OTUs of PSPNDH2, PSPNDH3, PSPNDH1, PSDH4, and PNDH5 was 1,730, 1,214, 1,606, 1,931, and 1,735, individually. The number of shared OTUs among PSPNDH2, PSPNDH3, PSPNDH1, PSDH4, and PNDH5 was172 (Fig. 1A). As for fungi, 3,519,792 high quality sequences were obtained with average sequences of 140,791, which were incorporated into 1,732 OTUs. PSPNDH2, PSPNDH3, PSPNDH1, PSDH4, and PNDH5 hold 605, 571, 502, 690, and 654 OTUs respectively, with unique OUTs of 233, 206, 171, 294, and 276, individually, and the shared OUTs of 159. In our research, the depth of sample sequencing increased, but the curve no longer increased significantly (Fig. S2). We believe that the amount of sequencing was sufficient, that is, the sample alpha diversity index has stabilized.

**Figure 2:**
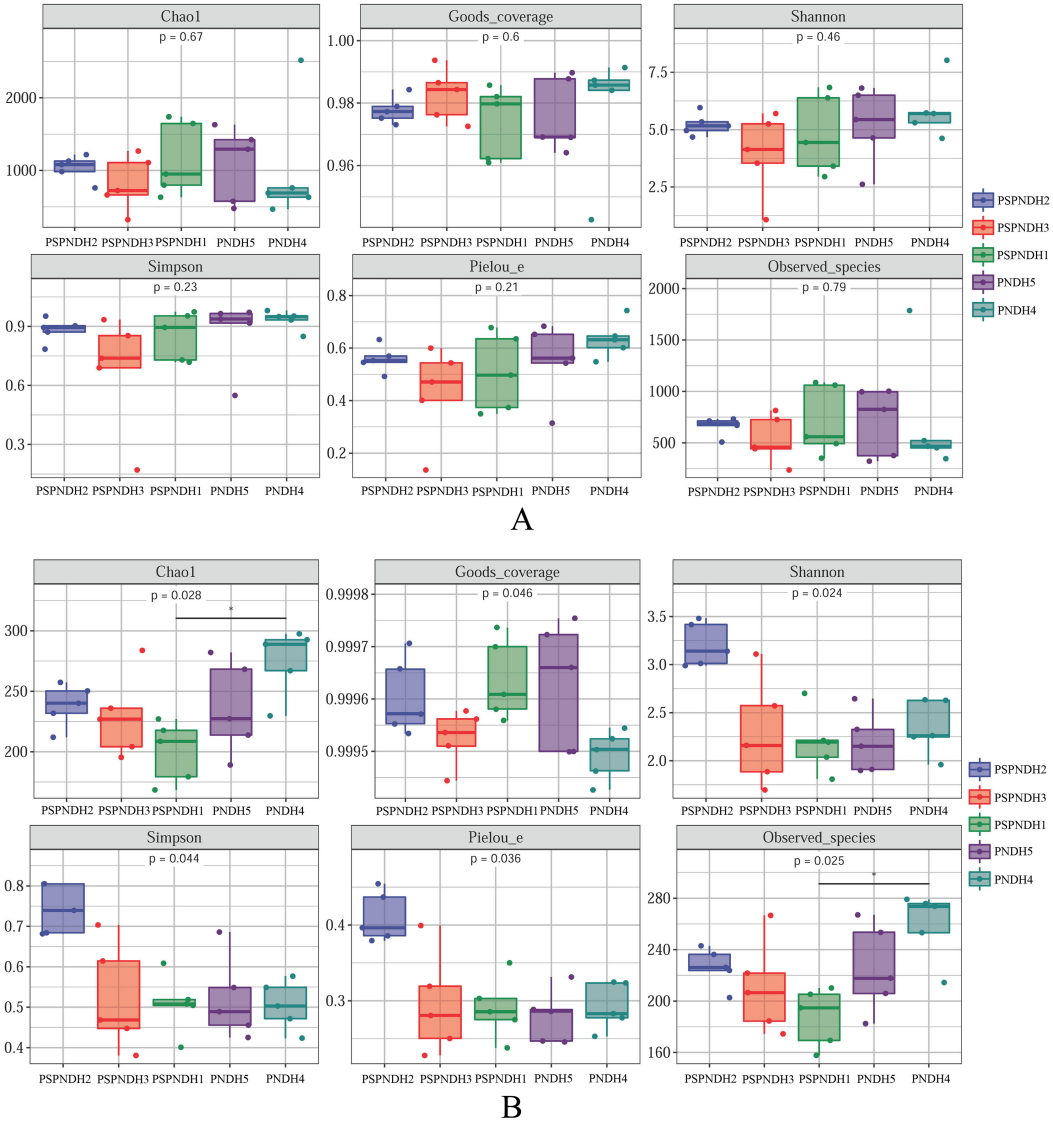
Phyllosphere bacterial (A) and fungal (B) community diversity. PSPNDH1: *P. simonii* × *P. nigra* cv. ‘ DH1’, PSPNDH2, *P. simonii* × *P. nigra* cv. ‘DH2’, PSPNDH3: *P. simonii* × *P. nigra* cv. ‘DH3’, PSDH4: *P. simonii* ‘DH4’, PNDH5: *Populus nigra* ‘DH5’.

No distinct differences of phyllosphere bacterial Chao1 index (*P* = 0.67), Goods_coverage (*P* = 0.60), Observed_species (*P* = 0.79), Pielou_e index (*P* = 0.21), Shannon index (*P* = 0.46), and Simpson index (*P* = 0.23) were observed among PSPNDH2, PSPNDH3, PSPNDH1, PSDH4, and PNDH5 (Fig. 2A). While, phyllosphere fungal Chao1 index (*P* = 0.028), Goods_coverage (*P* = 0.046), Observed_species (*P* = 0.025), Pielou_e index (*P* = 0.036), Shannon index (*P* = 0.024), and Simpson index (*P* = 0.044) observably differed among PSPNDH2, PSPNDH3, PSPNDH1, PSDH4, and PNDH5. PSDH4 hold the highest Chao 1 index of 275.15 and Observed_species of 259.22. PSPNDH2 owned the highest Pielou_e index, Shannon index, and Simpson index with 0.41, 3.21, and 0.74, respectively. Compared to PSPNDH2 and PSPNDH3, PSPNDH1 had the lowest Chao 1 index, Observed_species, Pielou_e index, Shannon index, and Simpson index with 200.26, 187.46, 0.29, 2.19, and 0.51, respectively (Fig. 2B).

### Phyllosphere microbial community composition

**Figure 3:**
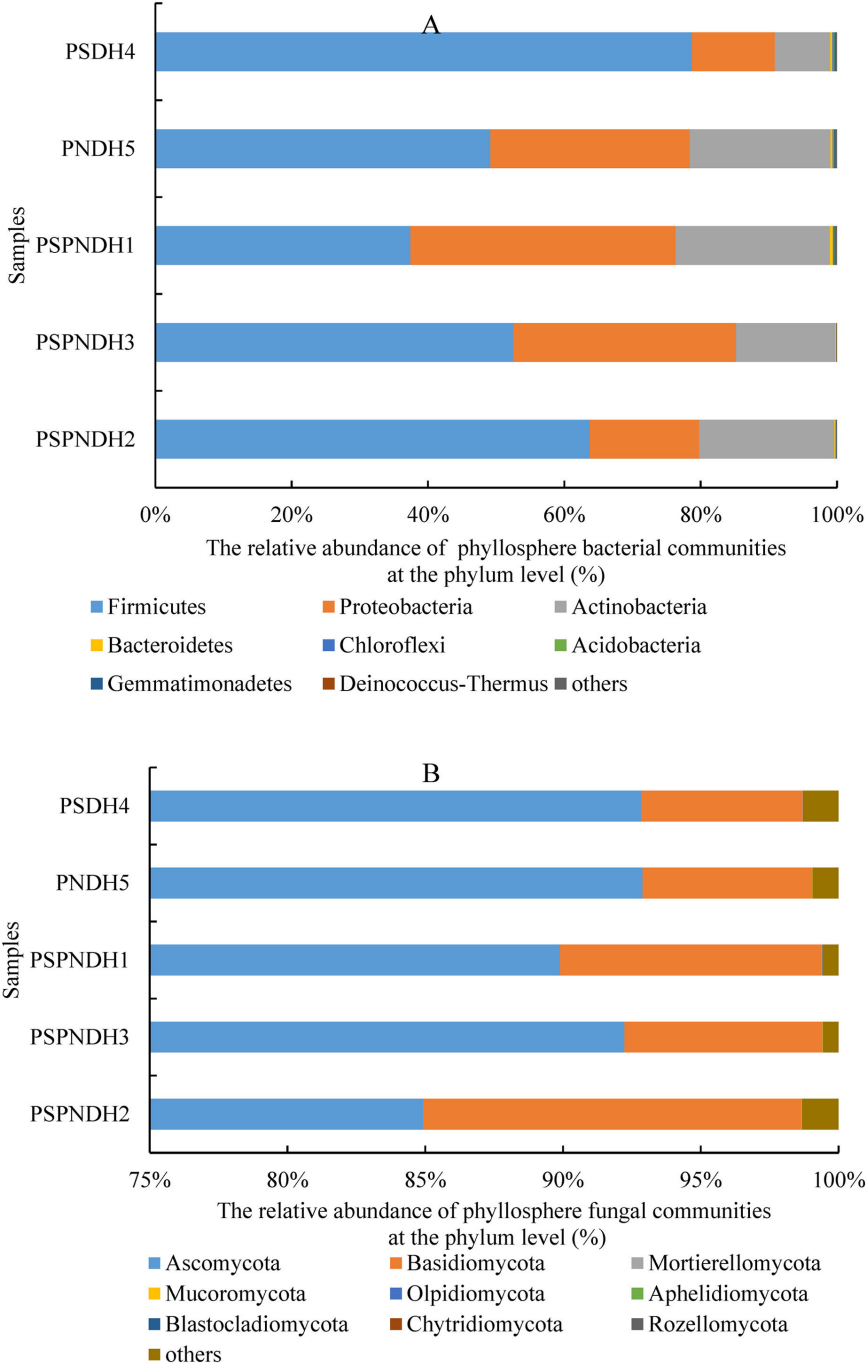
Phyllosphere bacterial (A) and fungal (B) community composition at the phylum level. PSPNDH1: *P. simonii* × *P. nigra* cv. ‘DH1’, PSPNDH2, *P. simonii* × *P. nigra* cv. ‘DH2’, PSPNDH3: *P. simonii* × *P. nigra* cv. ‘DH3’, PSDH4: *P. simonii* ‘DH4’, PNDH5: *Populus nigra* ‘DH5’.

In total, 35 bacterial phyla were detected across all 25 samples. The groups with average relative abundance more than 0.05% were Firmicutes, Proteobacteria, Actinobacteria, Bacteroidetes, Chloroflexi, Acidobacteria, Gemmatimonadetes, and Deinococcus-Thermus, accounting for 99.85%. The predominant groups were Firmicutes and Proteobacteria (Fig. 3A). At the class level, Bacilli and Gammaproteobacteria were two most dominant bacterial groups regardless of parent and the hybrid offspring (Fig. S3A). The relative abundance of Bacilli was relatively higher in PSDH4 (78.06%) compared to the other samples (37.04–63.55%). While, the relative abundance of Gammaproteobacteria showed the opposite trend, which was lower in PSDH4 (10.28%) than in the other samples (15.10–35.59%). At the genus level, 1,071 bacterial genera were obtained, and the bacterial groups with the average relative abundance more than 1% were *Exiguobacterium*, *Planomicrobium*, *Pseudomonas*, *Bacillus*, *Massilia*, *Frigoribacterium*, *Lysinibacillus*, *Planococcus*, *Pantoea*, *Curtobacterium*, and *Arthrobacter*, accounting for 71.26%.

In total, 9 fungal phyla, including Ascomycota, Basidiomycota, Mortierellomycota, Mucoromycota, Olpidiomycota, Aphelidiomycota, Blastocladiomycota, Chytridiomycota, and Rozellomycota were detected across all 25 samples (Fig. 3B). The predominant groups were Ascomycota, and Basidiomycota, accounting for 99.04%. At the class level, the phyllosphere fungal communities were dominated by Dothideomycetes (69.15–86.18%), Sordariomycetes (5.41–17.66%), Tremellomycetes (2.26–9.49%), and Agaricostilbomycetes (0.92–2.97%) (Fig. S3B). At the genus level, a total of 448 fungal genera were detected, and *Alternaria*, *Phialemoniopsis*, *Didymella*, *Filobasidium*, *Mycosphaerella*, and *Kondoa* were the dominant fungal communities, accounting for 90.10% (Fig. S3B).

**Figure 4:**
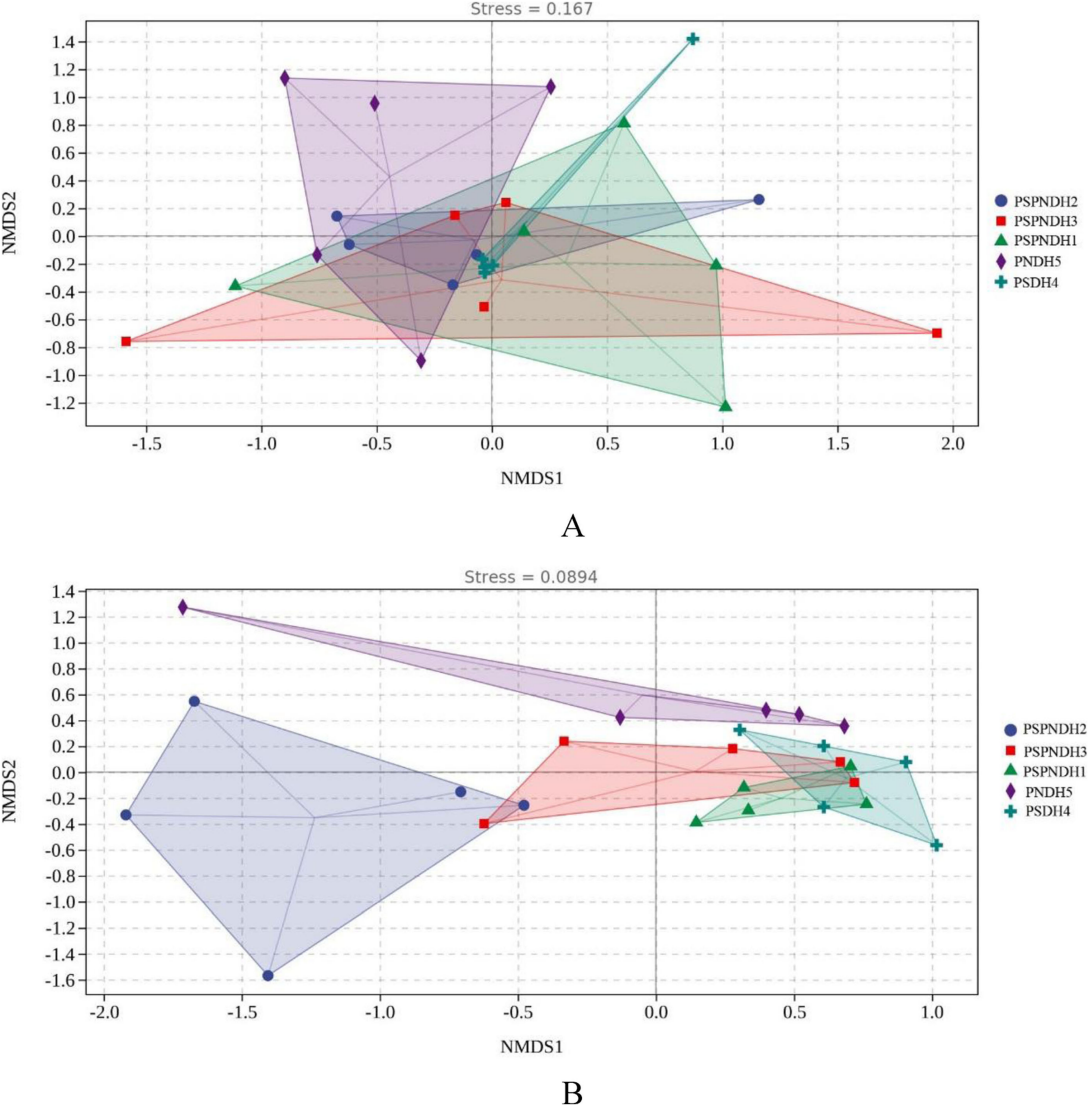
The NMDS of phyllosphere bacterial community (A) based on weighted-unifrac and fungal (B) community based on bray distance. PSPNDH1: *P. simonii* × *P. nigra* cv. ‘DH1’, PSPNDH2, *P. simonii* × *P. nigra* cv. ‘DH2’, PSPNDH3: *P. simonii* × *P. nigra* cv. ‘DH3’, PSDH4: *P. simonii* ‘DH4’, PNDH5: *Populus nigra* ‘DH5’.

**Figure 5:**
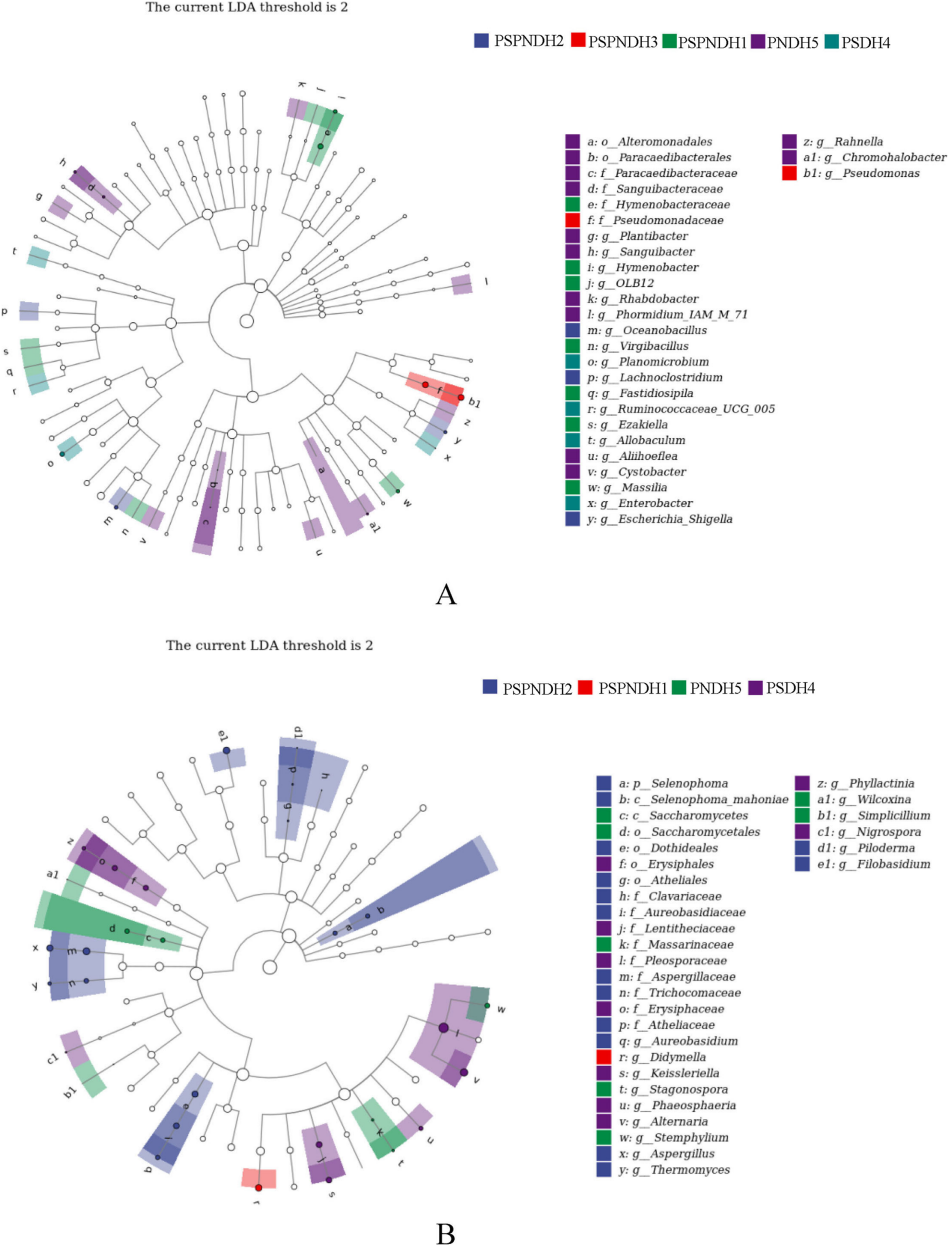
The LEfse of phyllosphere bacterial community (A) and fungal (B) community. PSPNDH1: *P. simonii* × *P. nigra* cv. ‘DH1’, PSPNDH2, *P. simonii* × *P. nigra* cv. ‘DH2’, PSPNDH3: *P. simonii* × *P. nigra* cv. ‘DH3’, PSDH4: *P. simonii* ‘DH4’, PNDH5: *Populus nigra* ‘DH5’.

The NMDS demonstrated that the phyllosphere microbial community composition obviously differed between PSDH4 and PNDH5, while, the phyllosphere microbial community composition presented some similarities under PSPNDH2, PSPNDH3, PSPNDH1, PSDH4, and PNDH5 (Fig. 4). In addition, PSPNDH2 enriched *Oceanobacillus*, *Lachnoclostridium*, *Escherichia_Shigella*, Selenophoma, Selenophoma_mahoniae, Dothideales, *Piloderma* and *Nigrospora*. PSPNDH3 enriched Pseudomonadaceae, *Pseudomonas*. PSPNDH1 enriched Hymenobacteraceae, *Hymenobacter*, *Massilia* and *Didymella*. PNDH5 enriched Alteromonadales, Paracaedibacterales, *Aliihoeflea*, *Cystobacter*, *Rahnella*, Saccharomycetes, Massarinaceae, *Stagonospora*, *Wilcoxina*, and *Simplicillium*. PSDH4 enriched *Planomicrobium*, *Allobaculum*, *Enterobacter*, Erysiphales, Lentitheciaceae, *Phaeosphaeria*, *Alternaria*, and *Phyllactinia* (Fig. 5).

### The relationships between leaf characteristics and phyllosphere microbial community

Phyllosphere bacterial Chao1 index (*r* = 0.63, *p* = 0.01; *r* = 0.64, *p* = 0.01) and Observed_species (*r* = 0.66, *p* = 0.01; *r* = 0.62, *p* = 0.02) had significantly positive correlation with soluble sugar and starch. Phyllosphere bacterial Pielou_e index (*r* = 0.55, *p* = 0.01), Shannon index (*r* = 0.62, *p* = 0.003), Simpson index (*r* = 0.49, *p* = 0.03) remarkably increased with the increase of soluble sugar content (Table 2). With regard to phyllosphere fungi, phyllosphere fungal Chao 1 index (*r* = 0.48, *p* = 0.01), Observed_species (*r* = 0.49, *p* = 0.01), and Shannon index (*r* = 0.39, *p* = 0.03) existed distinctly positive relation to soluble sugar (Table 2).

**Table 2:** The relationships between phyllosphere microbial community diversity with leaf characteristics.

	Soluble sugar	Starch
Bacteria		
Chao1 index	0.64**	0.65**
Goods_coverage	0.01	−0.21
Observed_species	0.66**	0.62**
Pielou_e index	0.55**	0.24
Shannon index	0.62**	0.35
Simpson index	0.49*	0.28
Fungi		
Chao1 index	0.48*	−0.00
Goods_coverage	−0.12	0.24
Observed_species	0.49*	−0.018
Pielou_e index	0.32	0.295
Shannon index	0.39	0.295
Simpson index	0.26	0.288

**Notes:**

* *P* < 0.05.

** *P* < 0.01.

The relative abundance of Firmicutes decreased with the increase of starch (*r* = −0.41, *p* < 0.05). While, the relative abundance of Actinobacteria (*r* = 0.55, *p* < 0.05), Bacteroidetes (*r* = 0.51, *p* < 0.05), Chloroflexi (*r* = 0.40, *p* < 0.05), Deinococcus-Thermus (*r* = 0.42, *p* < 0.05) increased with the increase of starch content (Table 3). The relative abundance of Basidiomycota hold significant positive correlation with starch (*r* = 0.40, *p* < 0.05) (Table 3).

**Table 3:** The relationships between phyllosphere microbial community composition at phylum level with leaf characteristics.

	Soluble sugar	Starch
Bacteria		
Firmicutes	−0.12	−0.41*
Proteobacteria	−0.06	0.28
Actinobacteria	0.33	0.55**
Bacteroidetes	0.23	0.51**
Chloroflexi	0.28	0.40*
Acidobacteria	0.29	0.37
Gemmatimonadetes	0.15	0.36
Deinococcus-Thermus	0.29	0.42*
Fungi		
Ascomycota	−0.26	−0.37
Basidiomycota	0.25	0.40*
Mortierellomycota	−0.03	0.32
Mucoromycota	0.23	0.39

**Notes:**

* *P* < 0.05.

** *P* < 0.01.

## Discussion

### Variation of plant functional traits of the parental clones and different hybrid progeny

Heterosis refers to the phenomenon that the F1 generation plants produced by crossing two parents with different genetic compositions are superior to the parents in one or more traits. The utilization of heterosis in modern breeding has achieved remarkable results in crops and trees, and most of the previous research results showed that the photosynthetic capacity of hybrid was significantly higher than that of their parents (Khan et al., 1998). In our study, the height and diameter at breast height of PSPNDH1, PSPNDH2, and PSPNDH3 existed higher than those of PSDH4, and PNDH5 (Table 1), which demonstrated that PSPNDH1, PSPNDH2, and PSPNDH3 owned higher plant biomass than those of PSDH4, and PNDH5. What’s more, leaf is one of the important vegetative organs of plants, and the research on leaf characteristics has always been an important part of poplar breeding research. A large number of studies have shown that there is a close relationship between leaves and growth, especially leaf area and leaf area index, which are closely related to biomass (Gebauer et al., 2016). Leaf area is also an important parameter to characterize the photosynthetic and transpiration capacity of plants and can be used as an early selection index for tree growth traits (Du et al., 2014; Guet et al., 2015). Therefore, it is very necessary to carry out the analysis of leaf-related traits in hybrid breeding. In our study, the leaf area and leaf moisture in PSPNDH1, PSPNDH2, and PSPNDH3 were significantly higher than those of PSDH4, and PSDH5 (Table 1), which proved that PSPNDH1, PSPNDH2, and PSPNDH3 had higher plant biomass from another perspective. In addition, PSDH1, PSDH2, and PSDH3 could increase the contents of starch, compared to PSDH4, and PSDH5 (Table 1), and all these results collectively demonstrated that the hybrid progeny had obvious heterosis.

### Variation of phyllosphere microganism of the parental clones and different hybrid progeny

As we all know, the leaf field of plants is a habitat characterized by a high degree of microbial diversity, but it is also a dynamic microenvironment due to direct exposure to a variety of abiotic and biological factors (Kinkel, 1997). In present study, we found that overall alpha diversity of the phyllosphere bacterial community existed no distinct differences among PSPNDH2, PSPNDH3, PSPNDH1, PSDH4, and PNDH5. While, phyllosphere fungal Chao1 index, Goods_coverage, Observed_species, Pielou_e index, Shannon index, and Simpson index observably differed among PSPNDH2, PSPNDH3, PSPNDH1, PSDH4, and PNDH5. PSPNDH2 had the highest Pielou_e index, Shannon index, and Simpson index. Our findings were not completely similar to the previous study from Liu et al. (2021) demonstrated that phyllosphere microbial community diversity of *P. cathayana* existed no distinct difference between male and female, which might be related to the similar physical and chemical properties, as well as similar physiological metabolism and traits of male and female leaves of *P. cathayana*, while male (PSDH4) and female (PNDH5) were not the same species in our present study. In addition, plant species and host plant genotype may be the main factor determining phyllosphere fungal community composition at the same location (Leveau, 2019).

In addition, the phyllosphere microbial communities are characterized by a small number of highly abundant taxa and a large number of relatively low abundance rare taxa (Vacher et al., 2016). At the phylum level, the dominant position of Proteobacteria in phyllosphere bacterial community has been confirmed by a large number of studies (Kembel et al., 2014). In our study, high-throughput sequence analysis of the 16S rDNA showed that Firmicutes, and Proteobacteria belonged to the predominant bacterial groups (Fig. 3A). At the class level, Bacilli and Gammaproteobacteria were the two most dominant bacterial classes regardless of parent and the hybrid offspring (Fig. S3A). The bacterial groups Alphaproteobacteria and Gammaproteobacteria have higher abundance and are typical representatives of Gram-negative bacteria in this habitat (Truchado et al., 2017; Izhaki et al., 2013; Huang et al., 2010). At the genus level, *Exiguobacterium*, *Planomicrobium*, *Pseudomonas*, *Bacillus*, *Massilia*, *Frigoriba*cterium, *Lysinibacillus*, *Planococcus*, *Pantoea*, *Curtobacterium*, *Arthrobacter* were the dominant microbial groups, accounting for 71.26%. Most of studies indicated that *Pseudomonas* and *Pantoea* are indigenous members of phyllosphere microbe (Ning et al., 2010), and they can produce a signal molecule called N-acyl-homoserine (Lv et al., 2013; Poonguzhali, Madhaiyan & Sa, 2007), that may help bacteria inhabit the harsh environment (Lv et al., 2013). Furthermore, *Pseudomonas* are considered to be very competitive plant leaf colonizers, and their extracellular polysaccharides and special pigments can protect them from ultraviolet radiation and osmotic stress (Qin et al., 2019), which was also frequently reported to benefit plant growth by means of its anti-phytopathogen ability (Alymanesh, Taheri & Tarighi, 2016; Mikiciński et al., 2016). The members of genus *Arthrobacter* can degrade a variety of organic pollutants and persist in the phyllosphere by up-regulating several genes known to contribute to epiphytic fitness (Scheublin et al., 2014). Collectively, these studies explained the existence of these bacteria in phyllosphere.

With regard to phyllosphere fungi, the predominant groups were Ascomycota, and Basidiomycota, accounting for 99.04%. At the class level, the dominant phyllosphere fungal communities were Dothideomycetes, Sordariomycetes, Tremellomycetes, and Agaricostilbomycetes (Fig. S3B). At the genus level, *Alternaria*, *Phialemoniopsis*, *Didymella*, *Filobasidium*, *Mycosphaerella*, and *Kondoa* were the dominant fungal communities. Interestingly, phyllosphere bacterial community and fungal community significantly differed between parental clones and hybrid progeny, while the phyllosphere microbial community presented some similarities under different hybrid progeny. This is in accordance with the view obtained from Maignien et al. (2014) indicated that a high variability of phyllosphere microbiomes across individual plants of the same species grown in the same location.

### The relationship between leaf characteristics and phyllosphere microbial community

Our study first provided a detailed insight into the effects of parental clones and different hybrid progeny on the above-ground microbial colonizers of plants. The host has greater genetic control over the phyllosphere microganism than the rhizosphere microbiota (Wagner et al., 2016). However, the rapidly fluctuating environmental conditions aboveground also play an important role, and only a few microbial groups that can adapt to harsh environments will flourish. The living environment of phyllosphere microorganisms is unstable and greatly affected by environmental changes (Jia et al., 2018), such as light, temperature and humidity (Verma, Ladha & Tripathi, 2001). In addition, plant species, leaf structural, chemical composition and secretions are known to influence phyllosphere microbial colonization (Leveau, 2019; Schlechter, Miebach & Remus-Emsermann, 2019; Espenshade et al., 2019) and phyllosphere microbial community diversity and composition (Hunter et al., 2010; Redford & Fierer, 2009; Kim et al., 2012). Our findings showed similar results, namely, that phyllosphere bacterial and fungal community diversity and composition had a significantly positive correlation with soluble sugar and starch (Tables 2 and 3), which was consistent with previous findings demonstrated that the phyllosphere fungal community structure was significantly affected by plant carbon (Jia et al., 2018). Thus, all these findings collectively established that plant traits are key factors that affect phyllosphere microbial community structure (Whipps et al., 2008).

## Conclusions

In conclusion, there were considerable variations of plant height, diameter at breast height, leaf area, length of petioles, leaf moisture content, and starch among different samples, and PSDH2 owned the highest plant height, diameter at breast height, and length of petioles. No distinct differences in phyllosphere bacterial community diversity were observed among PSPNDH2, PSPNDH3, PSPNDH1, PSDH4, and PNDH5, while phyllosphere fungal Chao1 index, Goods_coverage, Observed_species, Pielou_e index, Shannon index, and Simpson index observably differed among PSPNDH2, PSPNDH3, PSPNDH1, PSDH4, and PNDH5. PSPNDH2 had the highest fungal Pielou_e index, Shannon index, and Simpson index, and leaf characteristics contributed to the differentiation of phyllosphere bacterial and fungal communities between parents and hybrid offspring.

## Supplemental Information

10.7717/peerj.12915/supp-1 Supplemental Information 1 Plant functional traits.

10.7717/peerj.12915/supp-2 Supplemental Information 2 Supplemental Figures.

## Additional Information and Declarations

### Data Availability

The following information was supplied regarding data availability: The high-throughput sequencing raw data of phyllosphere and rhizosphere bacteria and fungi are available at SRA: PRJNA736812.
