# Supplementary material for: Study on the differences of phyllosphere microorganisms between poplar hybrid offspring and their parents
Source: PeerJ. 2022 Mar 15;10:e12915. doi: 10.7717/peerj.12915 (PMC8932310; doi:10.7717/peerj.12915)
Supplement: Supplemental Information 2 [file peerj-10-12915-s002.docx]

| PSPNDH1 |  | PNDH5 |  | PSDH4 |  | PSPNDH3 |  | PSPNDH2 |
| --- | --- | --- | --- | --- | --- | --- | --- | --- |
|  |  |  |  |  |  |  |  |  |
| PSPNDH2 |  | PSPNDH1 |  | PNDH5 |  | PSDH4 |  | PSPNDH3 |
|  |  |  |  |  |  |  |  |  |
| PSPNDH3 |  | PSPNDH2 |  | PSPNDH1 |  | PNDH5 |  | PSDH4 |
|  |  |  |  |  |  |  |  |  |
| PSDH4 |  | PSPNDH3 |  | PSPNDH2 |  | PSPNDH1 |  | PNDH5 |
|  |  |  |  |  |  |  |  |  |
| PNDH5 |  | PSDH4 |  | PSPNDH3 |  | PSPNDH2 |  | PSPNDH1 |

Figure S1 Site information. *P. simonii* × *P. nigra* cv. ‘DH1’, PSPNDH2, *P. simonii* × *P. nigra* cv. ‘DH2’, PSPNDH3: *P. simonii* × *P. nigra* cv. ‘DH3’, PSDH4: *P. simonii* ‘DH4’, PNDH5: *Populus nigra* ‘DH5’.


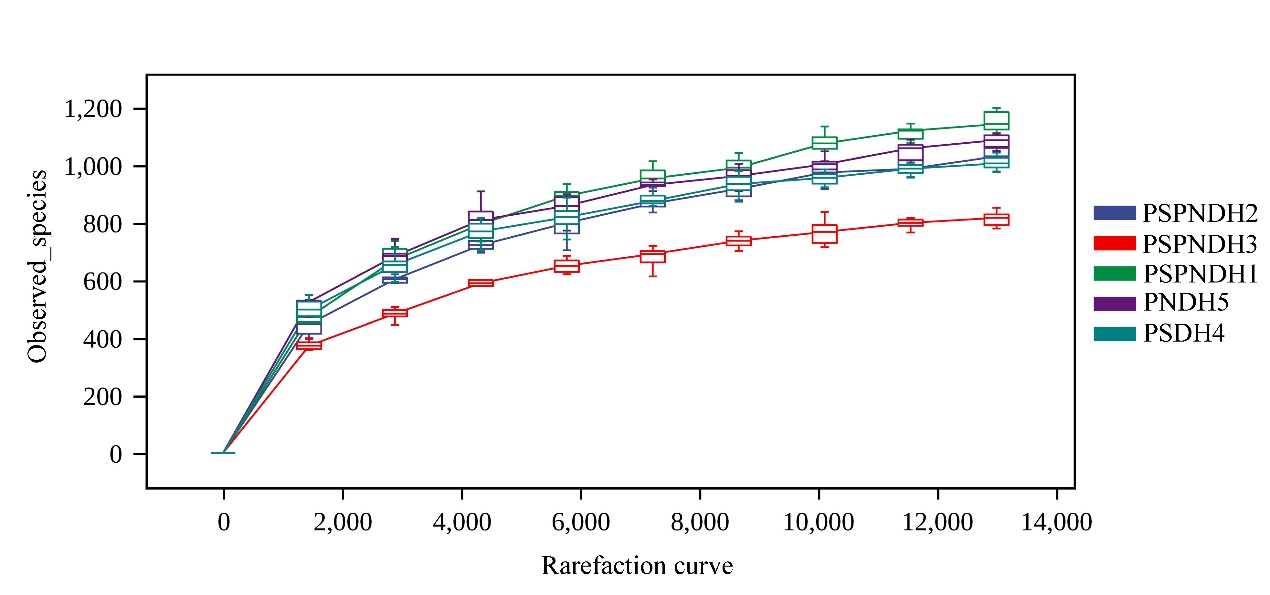


A


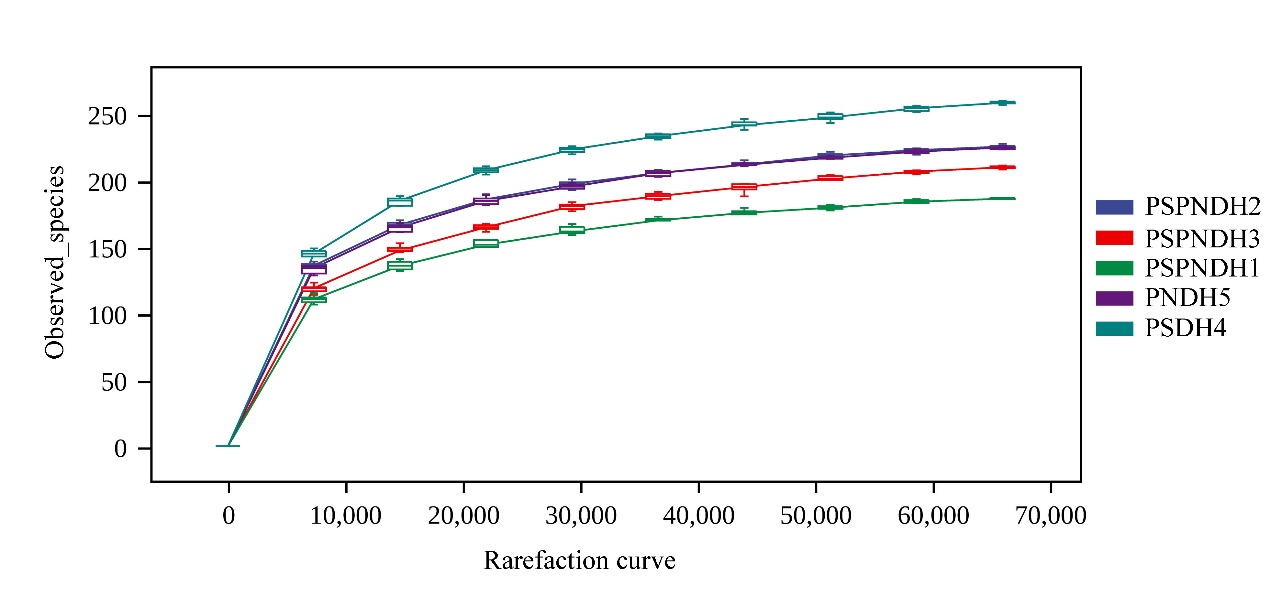


B

Figure S2 Phyllosphere bacterial (A) and fungal (B) species rarefaction curve. PSPNDH1: *P. simonii* × *P. nigra* cv. ‘DH1’, PSPNDH2, *P. simonii* × *P. nigra* cv. ‘DH2’, PSPNDH3: *P. simonii* × *P. nigra* cv. ‘DH3’, PSDH4: *P. simonii* ‘DH4’, PNDH5: *Populus nigra* ‘DH5’.

Figure S3 Phyllosphere bacterial (A) and fungal (B) community composition at the class level. PSPNDH1: *P. simonii* × *P. nigra* cv. ‘DH1’, PSPNDH2, *P. simonii* × *P. nigra* cv. ‘DH2’, PSPNDH3: *P. simonii* × *P. nigra* cv. ‘DH3’, PSDH4: *P. simonii* ‘DH4’, PNDH5: *Populus nigra* ‘DH5’.
